# A Ni(OH)_2_ nanopetals network for high-performance supercapacitors synthesized by immersing Ni nanofoam in water

**DOI:** 10.3762/bjnano.10.27

**Published:** 2019-01-25

**Authors:** Donghui Zheng, Man Li, Yongyan Li, Chunling Qin, Yichao Wang, Zhifeng Wang

**Affiliations:** 1 School of Materials Science and Engineering, Hebei University of Technology, Tianjin 300130, China; 2 School of Life and Environmental Sciences, Deakin University, Waurn Ponds, VIC 3216, Australia

**Keywords:** dealloying, Ni nanofoam, Ni(OH)_2_ nanopetals, metallic glass, supercapacitor

## Abstract

Developing a facile and environmentally friendly approach to the synthesis of nanostructured Ni(OH)_2_ electrodes for high-performance supercapacitor applications is a great challenge. In this work, we report an extremely simple route to prepare a Ni(OH)_2_ nanopetals network by immersing Ni nanofoam in water. A binder-free composite electrode, consisting of Ni(OH)_2_ nanopetals network, Ni nanofoam interlayer and Ni-based metallic glass matrix (Ni(OH)_2_/Ni-NF/MG) with sandwich structure and good flexibility, was designed and finally achieved. Microstructure and morphology of the Ni(OH)_2_ nanopetals were characterized. It is found that the Ni(OH)_2_ nanopetals interweave with each other and grow vertically on the surface of Ni nanofoam to form an “ion reservoir”, which facilitates the ion diffusion in the electrode reaction. Electrochemical measurements show that the Ni(OH)_2_/Ni-NF/MG electrode, after immersion in water for seven days, reveals a high volumetric capacitance of 966.4 F/cm^3^ at a current density of 0.5 A/cm^3^. The electrode immersed for five days exhibits an excellent cycling stability (83.7% of the initial capacity after 3000 cycles at a current density of 1 A/cm^3^). Furthermore, symmetric supercapacitor (SC) devices were assembled using ribbons immersed for seven days and showed a maximum volumetric energy density of ca. 32.7 mWh/cm^3^ at a power density of 0.8 W/cm^3^, and of 13.7 mWh/cm^3^ when the power density was increased to 2 W/cm^3^. The fully charged SC devices could light up a red LED. The work provides a new idea for the synthesis of nanostructured Ni(OH)_2_ by a simple approach and ultra-low cost, which largely extends the prospect of commercial application in flexible or wearable devices.

## Introduction

Nowadays, environmental contamination and energy crisis require new energy storage devices. This leads to a considerable interest in the research of supercapacitors because of their higher power density, longer cycling stability and faster charge/discharge periods compared to batteries [1–4]. Generally speaking, supercapacitors fall into two categories with different energy storage mechanisms. One is electrical double-layer supercapacitors (EDLCs) dominated by the electrostatic adsorption/desorption of electrolyte ions on the electrode surfaces. In EDLCs carbonaceous materials and their derivatives, such as active carbon, porous carbon, graphene, carbon nanotubes with good electrical conductivity and high specific surface area, are most commonly employed as electrode materials [5–7]. The other category are pseudocapacitors governed by reversible faradic redox reactions at the interface between active materials and electrolyte, for which transition metal oxides/hydroxides with multiple valence are used as electrode materials [8–9]. EDLCs hold a high power density and long cycling stability, but their practical application is limited by the low energy density. In comparison, pseudo-capacitors possess a higher energy density and are regarded as promising candidates for energy storage systems [10]. Among the various transition metal oxides/hydroxides, Ni(OH)_2_ is an ideal candidate for pseudo-capacitors due to its unique features such as high theoretical capacity and outstanding redox performance. Moreover, they are environmentally friendly, easily available and inexpensive [11–14]. However, bulk Ni(OH)_2_ is a semiconductor material with poor electrical conductivity [15], which leads to low capacity at a high scan rate and poor cycling stability. In order to overcome this shortcoming of bulk nickel hydroxide, various morphologies of Ni(OH)_2_ with a large specific surface area have been developed.

The conventional preparation method of Ni(OH)_2_ composite electrodes is to press a slurry of nickel hydroxide, conductive agents and binders on a conductive substrate (Ni foam, usually) [16–19]. However, this fabrication strategy is complicated and unsafely [20]. In addition, the presence of non-conductive binders not only increases the internal resistance but also the total mass of electrode, thus reducing the electrochemical performance. Therefore, the in situ synthesis of nickel hydroxide without binders has become a hot topic in recent years. For instance, Ni(OH)_2_ active materials have been loaded on stainless steel [21], carbon foam [22] and three-dimensional (3D) graphite foam [23]. Furthermore, 3D porous nickel materials have been used extensively as conductive substrate for electroactive Ni(OH)_2_ in supercapacitors because of the large surface area, good conductivity and compatibility with nickel hydroxide. Yuan et al. synthesized porous Ni(OH)_2_/NiOOH net on Ni foam by a chemical bath deposition and the electrode showed good rate capability [24]. Ke et al. demonstrated a nickel hydroxide@nanoporous gold/Ni foam electrode, which was synthesized by electrodeposition of a Sn–Au alloy on nickel foam with subsequent dealloying of Sn and electrodepostion of Ni(OH)_2_ on the nanoporous gold/Ni foam [25]. Liu et al. created Ni(OH)_2_/Cu_2_O nanosheets on nanoporous NiCu alloy surfaces by a hydrothermal method in H_2_O_2_ solution [26]. However, all above syntheses of nickel hydroxides require high temperature, nickel salts or/and oxidants that are toxic and hard to clean up. Moreover, the use of noble metals also increases the synthesis cost. To the best of our knowledge, there is no report on the in situ synthesis of nickel hydroxide nanosheets on Ni nanofoam through a simple and environmentally friendly method.

In the present work, we propose a simple and environmentally friendly two-step preparation, including the dealloying of Ni_40_Zr_20_Ti_40_ metallic glass in HF solutions and then immersing in deionized water for several days, to fabricate a binder-free, sandwich-like Ni(OH)_2_ nanopetals/Ni nanofoam/metallic glass (Ni(OH)_2_/Ni-NF/MG) electrode. Ni(OH)_2_ nanopetals interconnected with each other grow uniformly on the surface of the Ni nanofoam, which shortens the ions diffusion distance and facilitates the electrolyte transport. The as-synthesized Ni(OH)_2_/Ni-NF/MG electrodes demonstrate an excellent flexibility due to the ductile MG matrix and a good electrochemical performance. Moreover, the influence of immersion time in deionized water on the evolution of the Ni(OH)_2_ nanopetals and the specific capacitance of Ni(OH)_2_/Ni-NF/MG electrodes are investigated. Symmetric supercapacitor (SC) devices were also assembled and tested in this work.

## Experimental

### Synthesis

**Figure 1:**
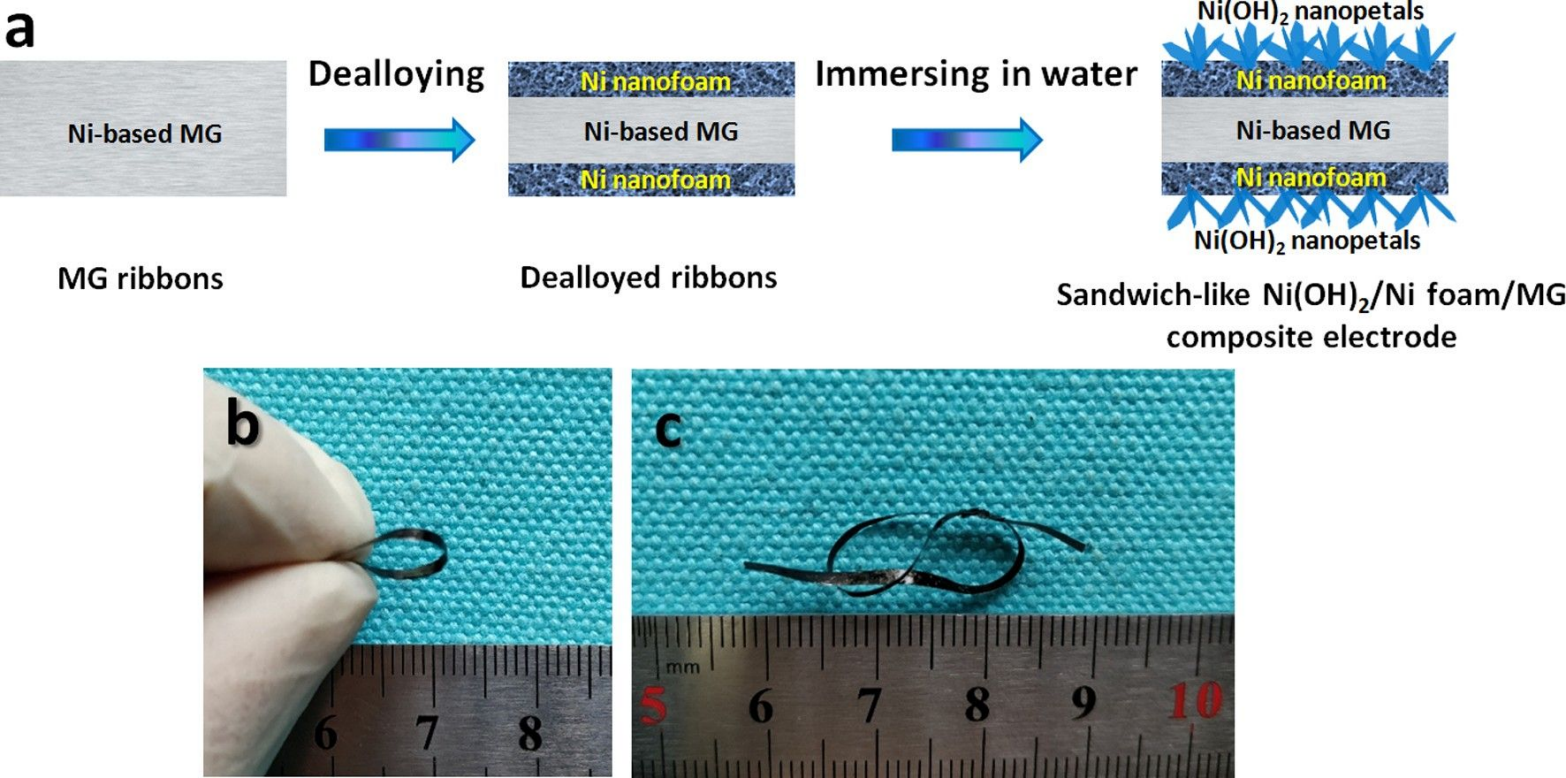
(a) Schematics of the fabrication process of the sandwich-like Ni(OH)_2_/Ni-NF/MG composite electrodes; (b, c) photographs of the as-fabricated electrodes with excellent flexibility.

A general synthesis scheme is depicted in Figure 1a. Firstly, the Ni_40_Zr_20_Ti_40_ (atom %) MG ribbons (2 mm wide and 20–30 µm thick) are fabricated by arc melting of the pure metals (99.99 wt %) followed by melt spinning [27–28]. Subsequently the dealloying process, as reported in our previous work [29–30], is carried out in 0.05 M HF solution for 4 h open to air at 298 K to form a Ni nanofoam layer on the MG surface. The dealloyed strips were washed with deionized water for three times and then immersed in deionized water for two, five or seven days at 298 K. Thereafter, the Ni(OH)_2_ nanopetals network was grown in situ on the Ni nanofoam. As a result of this growing process Ni(OH)_2_/Ni-NF/MG electrodes (ca. 2 mm wide and ca. 25 µm thick) were successfully obtained and were denoted as Ni(OH)_2_/Ni-NF/MG-2, Ni(OH)_2_/Ni-NF/MG-5, Ni(OH)_2_/Ni-NF/MG-7, respectively, according to the number of days immersed.

### Instrumental methods

The phase structure of the as-obtained composites was measured by X-ray diffraction (XRD, Bruker D8) with Cu Kα radiation. Chemical composition and valence state of the products were studied using X-ray photoelectron spectroscopy (XPS, Thermo Fisher Scientific). The surface morphology of the samples was characterized using a scanning electron microscope (SEM, Nova nanoSEM 450) equipped with an X-ray energy dispersive spectroscope (EDS) and a transmission electron microscopy (TEM, JEOL JEM-2100). The preparation process of the TEM sample was as follows: Firstly, the active materials were scraped off with a knife. Then the materials were dispersed in ethanol with ultrasonic vibration. Finally, the dispersed materials were dripped on copper mesh supported carbon film.

### Electrochemical measurements

The electrochemical tests of the Ni(OH)_2_/Ni-NF/MG electrodes were carried out in a standard three-electrode cell. The Ni(OH)_2_/Ni-NF/MG composites, a Pt net, and a Ag/AgCl electrode were employed as the working electrodes, the counter electrode and the reference electrode, respectively. Cyclic voltammograms (CV), galvanostatic charge/discharge curves (GCD) and electrochemical impedance spectroscopy (EIS) measurements were carried out using an electrochemical workstation (Chenhua CHI660D, China) in 1 M KOH aqueous solution at 298 K. The CV curves were examined in the voltage window of 0 to 0.5 V (vs Ag/AgCl) at scan rates of 2.5, 5, 10, 20, 50, 100 mV/s, and the GCD curves were collected at current densities of 0.5, 1, 2, 4, 8,12 A/cm^3^. The volumetric capacitance, based on the whole volume of the electrode including Ni-NF, MG and active materials, was calculated by the following equations according to GCD curves [31]:

[1] $$C_{\text{V}}=\frac{I\cdot \Delta t}{V\cdot \Delta V},$$

where *C*_v_ is the volumetric capacitance, Δ*t* is the discharge time, Δ*V* is the voltage range, *I* is the discharge current, and *V* is the nominal volume of the free-standing electrode. The volumetric capacitance can be also calculated by the following formula according to CV curves:

[2] $$C_{\text{V}}=\frac{\int_{\varphi_{\text{i}}}^{\varphi_{\text{f}}}i\text{d}\varphi }{V\cdot v\left(\varphi_{\text{f}}-\varphi_{\text{i}}\right)},$$

where φ_i_ and φ_f_ are initial and final potential, *i* is the current, dφ is the potential differential, and *v* is the scan rate of CV curves. EIS tests were performed by applying an AC voltage with 5 mV amplitude within a frequency range of 0.01 to 1000 kHz under open-circuit potential conditions.

### Assembly of symmetrical energy storage devices

The SC devices were assembled in a commercial 2032 button-cell shell, using Ni(OH)_2_/Ni-NF/MG-7 as active material, non-woven fabrics as separator and 1 M KOH as electrolyte under the pressure of 60 kg/cm^2^. The lengths of positive and negative ribbon are both of 15 cm in total. The SC device was tested by a two-electrode cell system. The CV curves were obtained at scan rates of 10, 20, 50 and 100 mV/s and the GCD curves were examined at current densities of 0.5, 1, 1.5, 2 A/cm^3^. The voltage of a fully charged SC device was further measured with a VC890C+ digital multimeter. The volumetric energy density (*E*_V_, Wh/cm^3^) and power density (*P*_V_, W/cm^3^) of a single SC device are calculated by the following equations:

[3] $$E_{\text{V}}=\frac{C_{\text{SC}}\cdot \Delta V_{\text{SC}}^{2}}{2\cdot 3600},$$

[4] $$P_{\text{V}}=\frac{3600\cdot E_{\text{V}}}{\Delta t_{\text{SC}}},$$

where *C*_sc_ is the specific capacitance, Δ*V*_sc_ is the potential range, and Δ*t* is the discharge time of a single SC device.

## Results and Discussion

### Material structure

The sandwich-like Ni(OH)_2_/Ni-NF/MG composite electrode inherits the excellent flexibility and ductility of Ni_40_Zr_20_Ti_40_ MG after dealloying in 0.05 M HF solutions for 4 h and being immersed in deionized water for two to seven days. As depicted in Figure 1b and Figure 1c, the as-synthesized Ni(OH)_2_/Ni-NF/MG electrodes can be bent into a small circle with a diameter of about 5 mm. Amazingly, it can be also tied into a small bowknot. Therefore, the as-prepared Ni(OH)_2_ nanopetals composite can be directly used as electrode for a supercapacitor without any binders.

**Figure 2:**
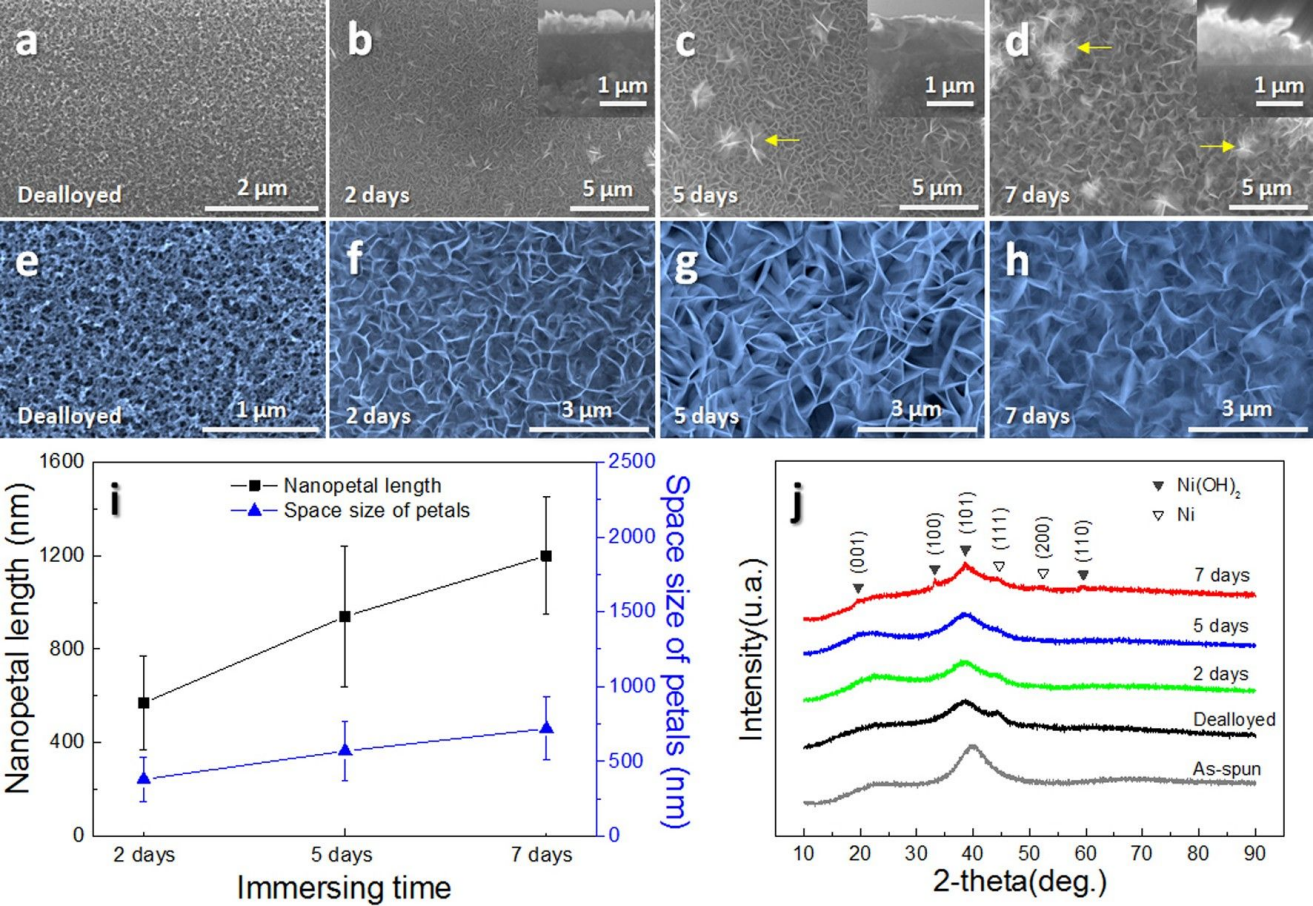
Low- and high-magnification SEM images of (a, e) the as-dealloyed ribbon, (b, f) Ni(OH)_2_/Ni-NF/MG-2, (c, g) Ni(OH)_2_/Ni-NF/MG-5, and (d, h) Ni(OH)_2_/Ni-NF/MG-7; the insets are the corresponding cross-sectional views. (i) Nanopetals length and space size of petals as functions of the immersion time. (j) XRD patterns of the as-spun, as-dealloyed and Ni(OH)_2_/Ni-NF/MG electrodes.

The morphologies of the as-dealloyed ribbons and as-prepared electrodes were examined by SEM, as shown in Figure 2a–h. The plane-view (Figure 2a) and the enlarged partial view (Figure 2e) show that the sample after dealloying possesses a 3D continuous pore structure with a ligament size of ca. 100 nm, which provides a path for the transportation of both electrons and ions in the electrolyte, resulting in the improved electrochemical performance. After immersing the dealloyed sample in water for two days, rose petal-like Ni(OH)_2_ with network structure are formed, as seen in Figure 2b. From Figure 2c,d, it is found that some flower-like structures (marked by arrows) composed of Ni(OH)_2_ nanopetals are formed upon the surface of the Ni(OH)_2_ nanopetals network, and are becoming larger with longer immersion time. These overgrowths of flower-like structures may arise from nucleation and coalescence processes [32]. The thickness of the nanopetals rises with immersion time from 0.5 μm (inset of Figure 2b) over 1.0 μm (inset of Figure 2c) to 1.5 μm (inset of Figure 2c). We can see from Figure 2f–h that the curly Ni(OH)_2_ nanopetals interweave and grow vertically on the surface of the Ni nanofoam, which forms an “ion reservoir”. In addition, length and spacing of Ni(OH)_2_ nanopetals grow with immersion time. The length of Ni(OH)_2_ nanopetals changes from ca. 600 to ca. 1200 nm and the spacing between them changes from ca. 300 to ca. 550 nm, as plotted in Figure 2i. This structural characteristic of an “ion reservoir” would bring about fast ion/electron transfer, short ion transport distances and sufficient contact at active material/electrolyte interfaces, which might improve the electrochemical performance [33].

Figure 2j shows typical XRD patterns of the as-spun ribbon, the as-dealloyed ribbon and the as-synthesized Ni(OH)_2_/Ni-NF/MG composites immersed in deionized water for different days. The original ribbon presents a characteristic broad halo peak of metallic glass without appreciable sharp crystalline peaks, indicating the formation of a single homogeneous metallic glassy structure. After dealloying, in addition to a broad halo peak, two peaks located at 44.5° and 51.8° can be assigned to the (111) and (200) planes of Ni metal (JCPDS no. 04–0850), respectively. This further confirmed the formation of the Ni nanofoam layer after dealloying of MG precursors. The other diffraction peaks at 19.2°, 33.1°, 38.5°, 59.1° can be attributed to the (001), (100), (101) and (110) planes of β-Ni(OH)_2_ (JCPDS no. 14–0177), respectively, suggesting the successful synthesis of Ni(OH)_2_ nanopetals upon the Ni nanofoam surface after immersing in deionized water for several days.

**Figure 3:**
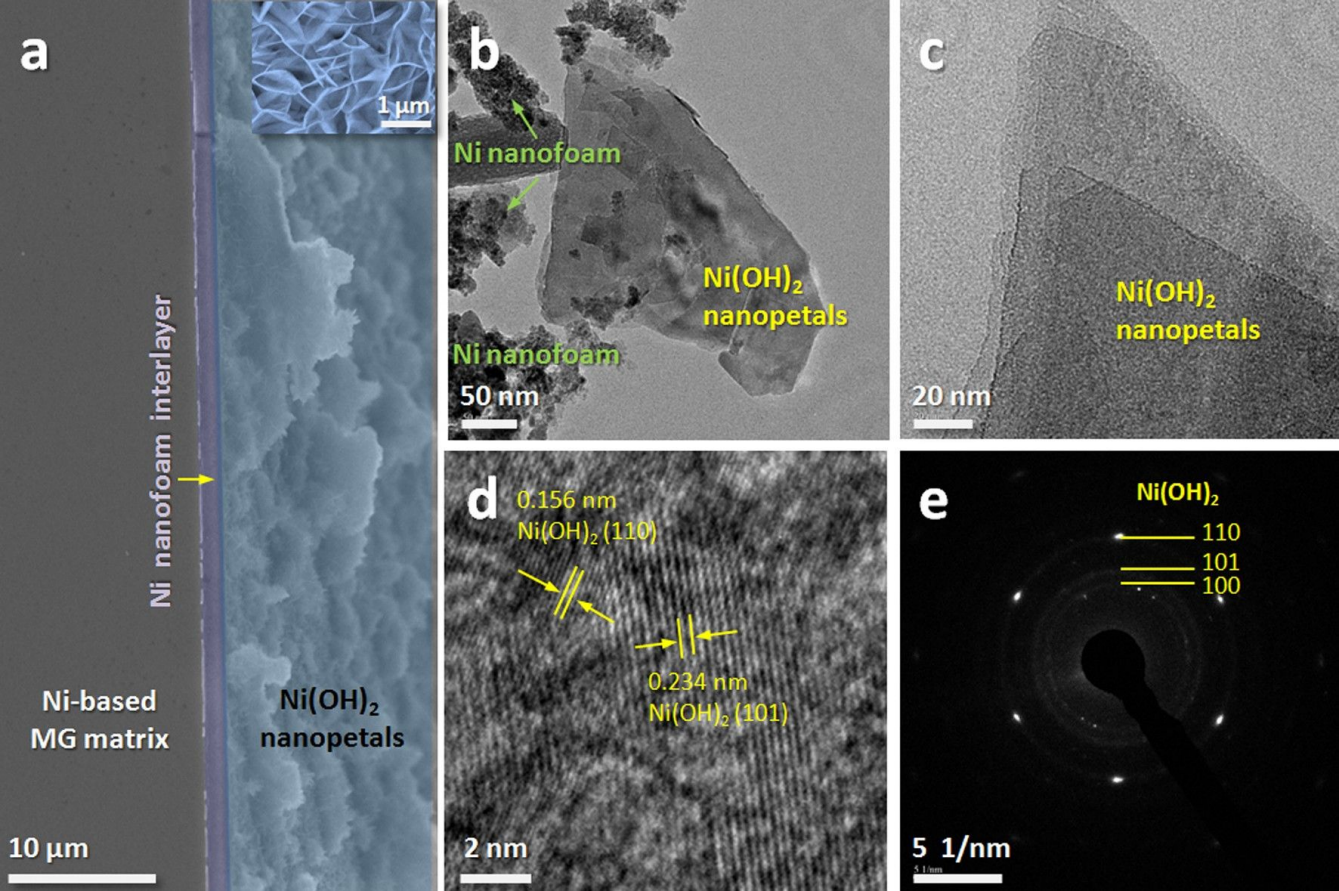
(a) Cross-sectional SEM images of the Ni(OH)_2_/Ni-NF/MG-5, the inset is a local high-resolution image; (b, c) low- and high-magnification TEM images; (d) HRTEM image; (e) SAED pattern of Ni(OH)_2_ nanopetals.

To understand the structure of the as-obtained electrode, the cross-sectional view of the sample immersed in deionized water for five days is investigated (Figure 3a). The sandwich-like Ni(OH)_2_ composite electrode is successfully prepared after initially dealloying the MG precursor and subsequently immersing the dealloyed sample in deionized water. The layers of the structure are in close contact with each other, indicating the good integrity of the electrode. The inset is a locally magnified SEM image showing the structure of the “ion reservoir” and is in accordance with the SEM images in Figure 2c and Figure 2g. Morphology and structure of as-synthesized Ni(OH)_2_ nanopetals are further observed by TEM. As seen from Figure 3b, the intersected nanopetals are loaded on the Ni nanofoam. Figure 3c shows the high electron transparency of the nanopetals with several layers stacked together, indicating an ultrathin nature [12,34]. The high-resolution TEM image in Figure 3d provides more detailed lattice structure information of the Ni(OH)_2_ nanopetals. The interplanar distances of 0.156 nm and 0.234 nm are related to the (110) and (101) planes of the Ni(OH)_2_, respectively. The SAED pattern taken from a single nanopetal shown in Figure 3e indicates that the Ni(OH)_2_ nanopetals are polycrystalline. The diffraction rings belong to the (100), (101), and (110) planes of Ni(OH)_2_, respectively, which is consistent with the XRD results and can further confirm the successful synthesis of Ni(OH)_2_ nanopetals. It is worth noting that the sandwich-like Ni(OH)_2_/Ni-NF/MG composite electrode exhibits excellent flexibility. This can be explained by the fact that the Ni-NF/MG substrate was obtained by dealloying ductile Ni_40_Zr_20_Ti_40_ MG. The cross section (Figure 3a) shows that the Ni-NF layer smoothly adheres to the MG matrix. The thickness of the Ni-NF layer is ca. 1.5 μm; the Ni_40_Zr_20_Ti_40_ MG is ca. 22 μm thick. It is well known that Ni-based MGs exhibit a much larger elasticity and ductility than their corresponding crystalline alloys. A sufficiently thick MG layer acts as a ductile support to the dealloyed Ni-NF/MG substrate. In additon, the in situ growth in deionized water offers a good connection between Ni-NF and Ni(OH)_2_.

### The growth mechanism of Ni(OH)_2_ nanopetals

According to the SEM images (Figure 2b–d), Ni(OH)_2_ nanopetals and flower-like microspheres grow on the surface of Ni-NF/MG after immersion in water. A possible growth mechanism of Ni(OH)_2_ nanopetals is proposed in the following. A large amount of defects (twin boundaries, stack faults and/or dislocations) were generated in the nanoporous metal ligament surface after dealloying [35]. When the nanoporous Ni (Ni-NF) with many defects with higher distortion energy was placed in deionized water, many microgalvanic cells were formed at the surface of Ni-NF. Most likely, the following electrode reactions of a microgalvanic cell take place [36]:

[5] $$\text{Anode: }2\text{Ni}\to 2{\text{Ni}}^{2+}+4{\text{e}}^{-},$$

[6] $${\text{Cathode: O}}_{\text{2}}+2{\text{H}}_{\text{2}}\text{O}+4{\text{e}}^{-}\to 4{\text{OH}}^{-},$$

[7] $$\text{Cell reaction: }2\text{Ni}+{\text{O}}_{2}+2{\text{H}}_{\text{2}}\text{O}\to 2\text{Ni}{\left(\text{OH}\right)}_{2}.$$

Based on the above reactions, Ni(OH)_2_ crystals nucleated and grew at the surface of 3D Ni-NF ligaments. The morphological change of Ni(OH)_2_ nanopetals after different immersion times was shown in Figure 2. The pH value of deionized water is about 7.0. Research has shown that when the pH value is higher, the crystals tend to grow into a flake structure in the [100] or [010] directions [37]. The anisotropic growth leads to the formation of high-density crosslinked nanopetals. The flower-like structures composed of Ni(OH)_2_ nanopetals are formed through Ostwald ripening [38]. Meanwhile, the dissolved tiny and unstable plates provide the source material for the growth of plates during the dissolution and recrystallization process. The dissolved nickel atoms may continuously attach and bond to the surface of larger nanopetals, and form flower-like structures in order to achieve a minimum total free energy.

### Chemical characteristics of the composite surface

**Figure 4:**
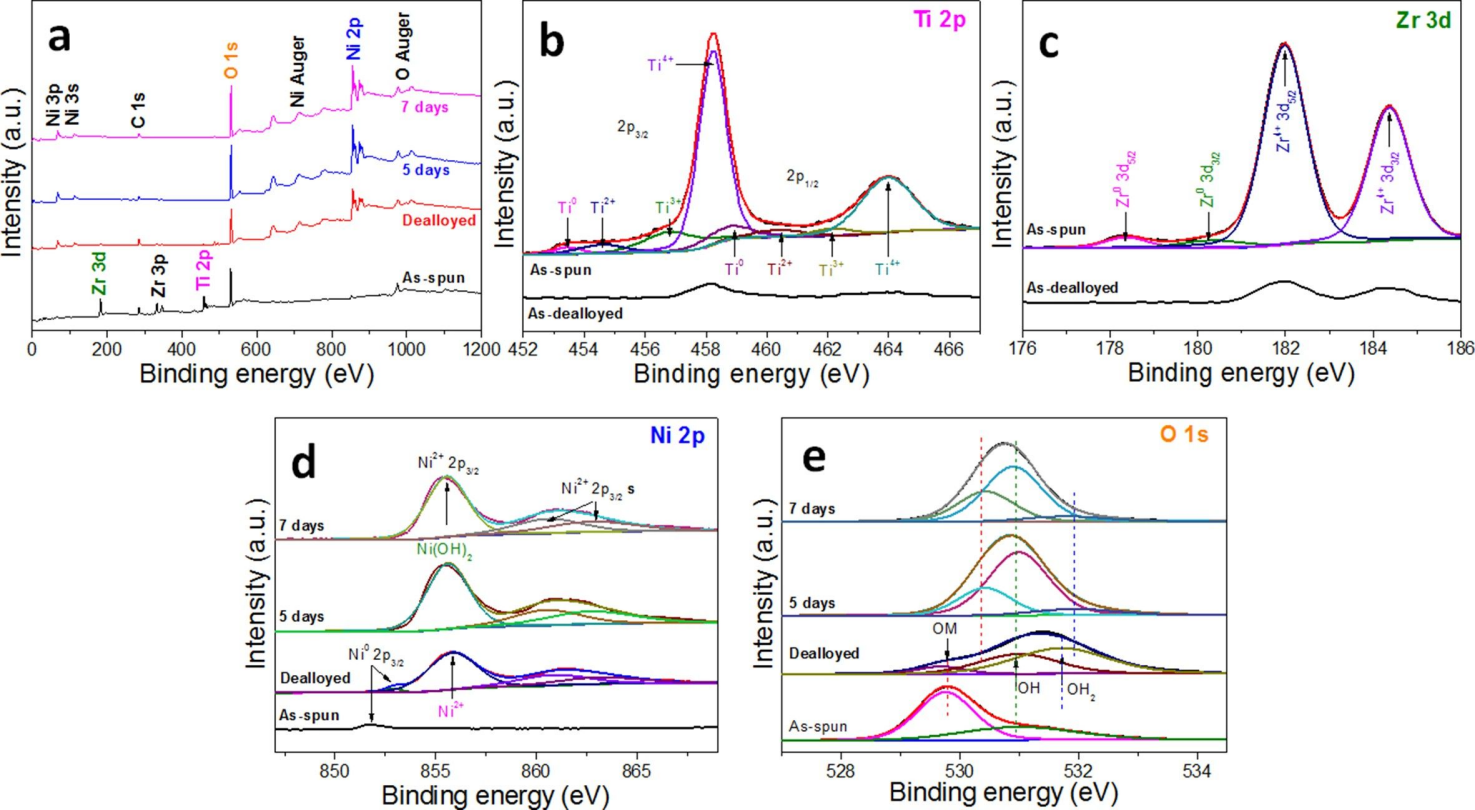
XPS spectra of the elements of the as-spun ribbon, as-dealloyed ribbon and as-synthesized electrode: (a) survey spectrum, (b) Ti 2p, (c) Zr 3d, (d) Ni 2p and (e) O 1s.

In order to clarify the changes in chemical state of the elements, XPS measurements are performed for the as-spun, as-dealloyed, and as-synthesized Ni(OH)_2_ specimens. Figure 4a shows that the XPS spectra over a wide energy region exhibit the main peaks of Zr 3d, Ti 2p, O 1s, and C 1s for the as-spun Ni-Zr-Ti alloy, while large peaks of Ni 2p and O 1s appear for both the as-dealloyed alloy and as-synthesized Ni(OH)_2_. It is found that the intensity of O 1s peak greatly increases for the as-dealloyed alloy immersed in deionized water for five and seven days. The deconvolution results of the Ti 2p and Zr 3d spectra measured for the as-spun and as-dealloyed alloy, respectively, are shown in Figure 4b and Figure 4c, respectively. For the as-spun alloy, it is found that the large peaks correspond to Ti^4+^ and Zr^4+^ on the alloy surface, whereas the minor peaks are associated to lower oxidation states. These phenomena can be explained through a preferential oxidation of Ti and Zr during the alloy fabrication and the subsequent oxidation exposed to the air. After immersion in 0.05 M HF for 4 h, the signals for Ti and Zr become very weak due to the dissolution of Ti and Zr. It is clarified that the surface film of as-spun alloy mainly consists of Ti^4+^ and Zr^4+^ oxides. On the other hand, Figure 4d and Figure 4e reveal the Ni 2p_3/2_ and O 1s peaks obtained from the as-spun alloy and as-dealloyed alloy before and after immersion in deionized water. The O 1s region analyzed by using a Gaussian fitting method (Figure 4e) shows three chemical states of oxygen. The strong peak at 531.1 eV is related to bound hydroxide groups (OH^−^) and the peak at 529.9 eV is assigned to a typical metal–oxygen bond (O–M). Additionally, the peaks at 532.1 eV can be ascribed to water adsorbed at the material surface [39]. For the as-dealloyed sample before and after immersion in water, the Ni 2p_3/2_ spectrum (Figure 4d) consists of a major characteristic peak at 855.8 eV corresponding to Ni(OH)_2_ and two satellite peaks (indicated as s.), which is in good agreement with previous reports [40–41]. It should be mentioned that the Ni hydroxide peak increases greatly with longer immersion times, indicating that the immersion facilitates the in situ growth of Ni(OH)_2_ on the Ni nanofoam surface.

### Supercapacitor performance

**Figure 5:**
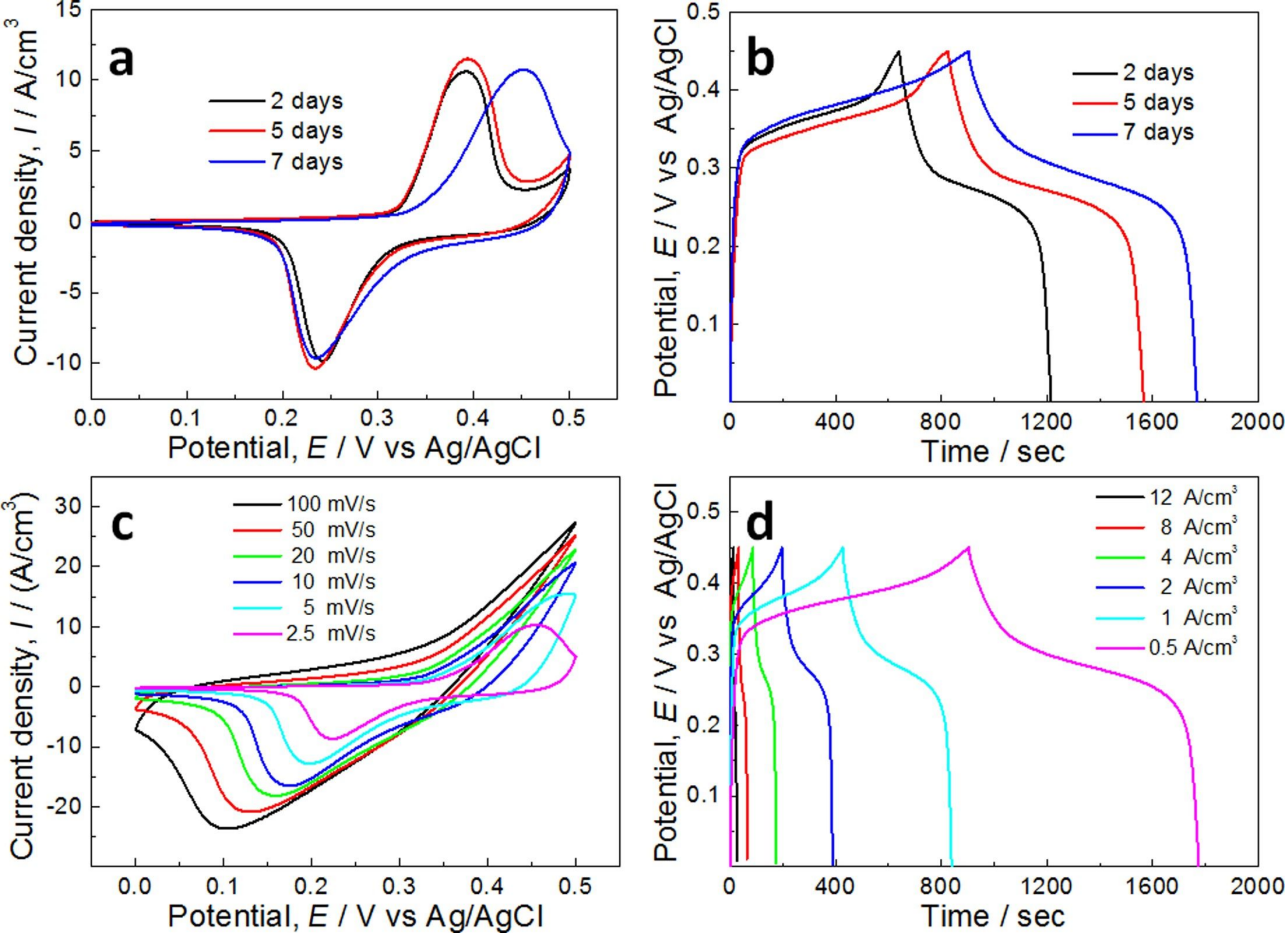
(a) CV curves of the Ni(OH)_2_/Ni-NF/MG-2, Ni(OH)_2_/Ni-NF/MG-5, Ni(OH)_2_/Ni-NF/MG-7 electrodes at a scan rate of 0.5 mV/s in 1 M KOH solution; (b) GCD curves of the Ni(OH)_2_/Ni-NF/MG-2, Ni(OH)_2_/Ni-NF/MG-5, Ni(OH)_2_/NiNF/MG-7 at a current density of 0.5 A/cm^3^; (c) CV curves of the Ni(OH)_2_/Ni-NF/MG-5 at different scan rates from 2.5 to 100 mV/s; (d) GCD curves of the Ni(OH)_2_/Ni-NF/MG-5 at different current densities from 0.5 to 12 A/cm^3^.

The electrochemical performance of the Ni(OH)_2_ nanopetals on Ni nanofoam was evaluated systematically by CV and GCD measurements with a three-electrode cell in 1 M KOH aqueous solution at 298 K (Figure 5). The CV curves of the Ni(OH)_2_/Ni-NF/MG-2, Ni(OH)_2_/Ni-NF/MG-5, and Ni(OH)_2_/Ni-NF/MG-7 electrodes at the scan rate of 2.5 mV/s with the potential window from 0 to 0.5 V are shown in Figure 5a. A set of strong redox peaks can be clearly detected for the three electrodes, which corresponds to reversible reactions of Ni^2+^ ↔ Ni^3+^. The redox peaks illustrate that pseudo-capacitive behavior occurred at the electrode/electrolyte interface. The corresponding kinetically reversible faradic redox reaction involved in the electrochemical process is [25,42]:

[8] $$\text{Ni}{\left(\text{OH}\right)}_{2}+{\text{OH}}^{-}↔\text{NiOOH}+{\text{H}}_{\text{2}}\text{O}+{\text{e}}^{-}.$$

The specific capacitance of Ni(OH)_2_/Ni-NF/MG-2, Ni(OH)_2_/Ni-NF/MG-5 and Ni(OH)_2_/Ni-NF/MG-7 calculated from the CV curves are 728.1, 823.8 and 866.3 F/cm^3^, respectively. It is notable that the anodic peak of the Ni(OH)_2_/Ni-NF/MG-7 electrode is shifted to a more positive value than the other two peaks, which is related to the poor conductivity (larger ohmic resistance) of Ni(OH)_2_ nanopetals [21,43]. Larger ohmic resistance leads to slow kinetics of charge transport and interfacial charge transfer of the material. Thus, the electrode reaction rate slows down, resulting in a reduced reversibility of the redox processes. This is consistent with the SEM images (Figure 2d and Figure 2h) of the samples immersed in deionized water for seven days showing the thickest Ni(OH)_2_ nanopetals layer among the experimental samples. The GCD curves measured at current density of 0.5 A/cm^3^ for the three Ni(OH)_2_/Ni-NF/MG electrodes further elucidate the pseudo-capacitance characteristics, as shown in Figure 5b. Every GCD curve has an obvious charge–discharge plateau and the voltage position is in agreement with the CV curves, demonstrating that the faradic redox reactions mainly contribute to the capacitance. The specific capacitance values of the electrodes were calculated from Equation 1. The Ni(OH)_2_/Ni-NF/MG-7 electrode presented the highest volumetric capacitance value of 966.4 F/cm^3^. It is worth noting that the real value of the volumetric capacitance should be much higher, because the present volumetric capacitance is calculated by the total volume of the sandwich-structured electrode, and the volume of active Ni(OH)_2_ accounts for only a small proportion of the whole electrode. Figure 5c discloses the CV response of Ni(OH)_2_/Ni-NF/MG-7 at different scan rates ranging from 2.5 to 100 mV/s. The results indicate that the current response increases with an increase of the scan rate. Moreover, it is found that the reduction peaks are shifted to more negative values with the increase of the scan rate, whereas the anodic peaks are shifted to more positive values and finally disappear at scan rates above 20 mV/s. This phenomenon is related to electrochemical polarization, i.e., the electron flow rate not keeping pace with the electrode reaction [44]. The GCD (Figure 5d) was recorded at different current densities from 0.5 to 12 A/cm^3^. The nonlinear GCD curves confirm typical pseudo-capacitive behavior, which is also in accord with Figure 5c.

**Figure 6:**
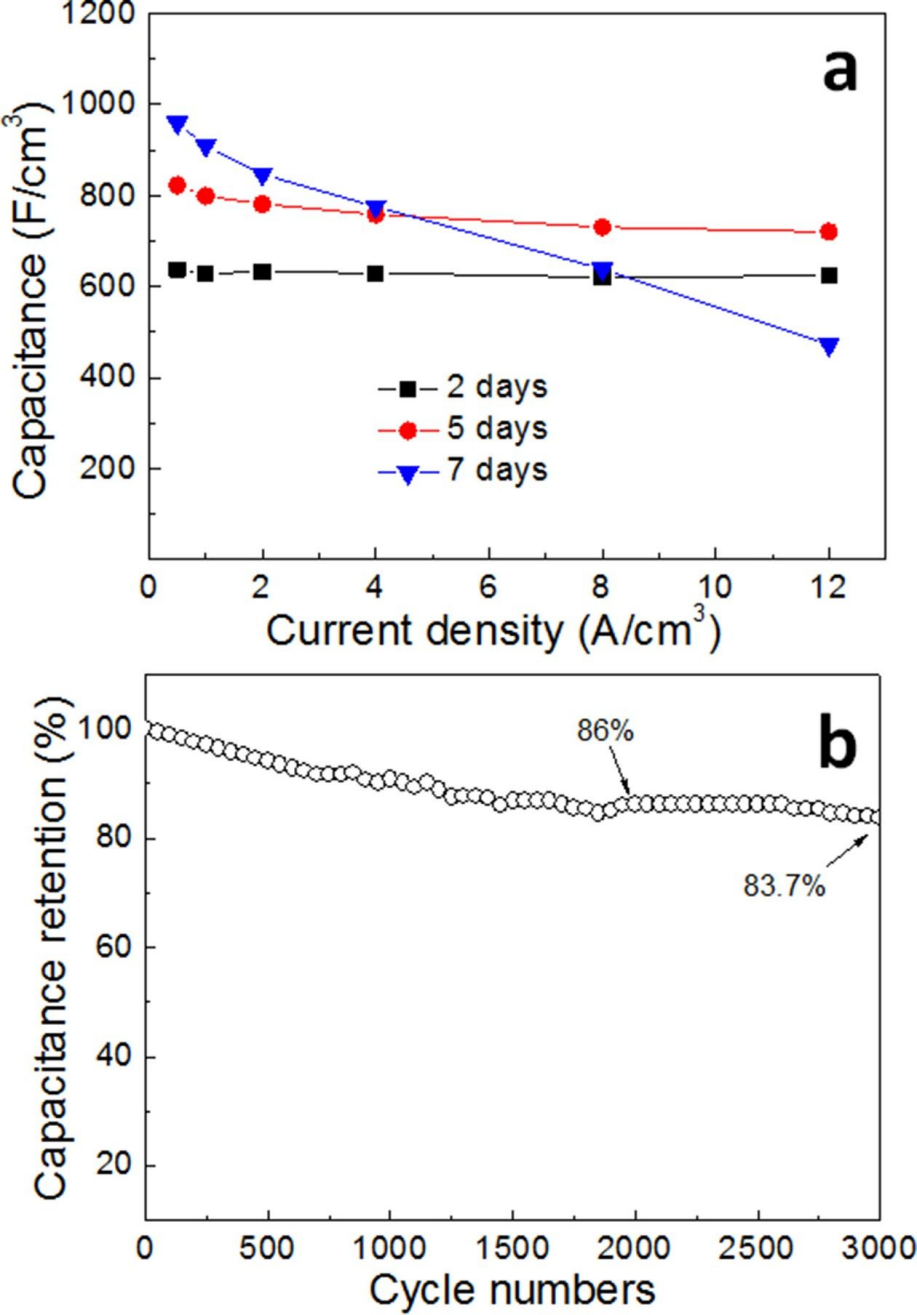
(a) Volumetric capacitance of the Ni(OH)_2_/Ni-NF/MG-2, Ni(OH)_2_/Ni-NF/MG-5, Ni(OH)_2_/Ni-NF/MG-7 electrodes at different discharge current densities; (b) volumetric capacitance versus cycle number of Ni(OH)_2_/Ni-NF/MG-5 at a galvanostatic charge and discharge current density of 1 A/cm^3^.

Figure 6a shows the volumetric capacity of the three electrodes obtained from the GCD curves at different current densities according to Equation 1. As we can see, the volumetric capacitance of the three electrodes decreases when the current density is changed from 0.5 to 12 A/cm^3^. This is the results of less electroactive materials being available because of limited ion diffusion when the discharge current density increases [45]. The volumetric capacitance values of Ni(OH)_2_/Ni-NF/MG-7 are found to be 966.4, 915.1, 852.4, 761.8, 568.9 and 328 F/cm^3^ at discharge current densities of 0.5 1, 2, 4, 8 and 12 A/cm^3^ (retention ratio 34%), respectively, whereas for the Ni(OH)_2_/Ni-NF/MG-5 electrode, it is 822.6, 798.7, 781.3, 758.2, 730.7 and 720 F/cm^3^ (retention ratio 87.5%), respectively, and for Ni(OH)_2_/Ni-NF/MG-2, it is 637.7, 628.9, 632.0, 629.3, 620.4 and 624.0 F/cm^3^ (retention ratio 97.9%), respectively. Obviously, the rate performance of the Ni(OH)_2_/Ni-NF/MG-2 electrode is the best, while that of Ni(OH)_2_/Ni-NF/MG-7 is the worst. That is probably because the structural differences of the surface Ni(OH)_2_. When the immersion time is short, the Ni(OH)_2_ nanopetals exhibit a network shape. With increasing immersion time, clusters of nanopetals accumulate into a flower morphology upon the Ni(OH)_2_ network layer, resulting in a thickness increase of the Ni(OH)_2_ layer and a decline in conductivity. A higher conductivity of the electrode leads to a better rate performance. Good conductivity makes the electrode stand up the impact of higher currents. This is due to the fast electron transfer occurring at high current densities through which the minimum specific capacitance is reduced when compared to its initial value [46]. Cycle performance is another key factor affecting the practical application of electrode materials. The cycling stability (Figure 6b) of the Ni(OH)_2_/Ni-NF/MG-5 electrode is evaluated by a continuous GCD test up to 3000 cycles at a current density of 1 A/cm^3^. It is noticed that the capacitance of Ni(OH)_2_/Ni-NF/MG-5 decreases gradually without an obvious activation process. The Ni(OH)_2_/Ni-NF/MG-5 delivers a relatively high specific capacitance of 687.7 F/cm^3^ with 86.1% retention of its initial capacitance after 2000 cycles, and even shows an excellent cycling stability of 83.7% after 3000 cycles. Although the capacitance retention of Ni(OH)_2_/NiNF/MG-5 is not comparable to those of a 2D MoSe_2_-Ni(OH)_2_ nanohybrid electrode (90% retention after 3000 cycles at 2 A/g) [46] and a NiCo-LDH composite electrode (90% retention after 5000 cycles at 20 mA/cm^2^) [47], it exceeds most of the Ni(OH)_2_@Ni foam electrodes [14,42,48].

**Figure 7:**
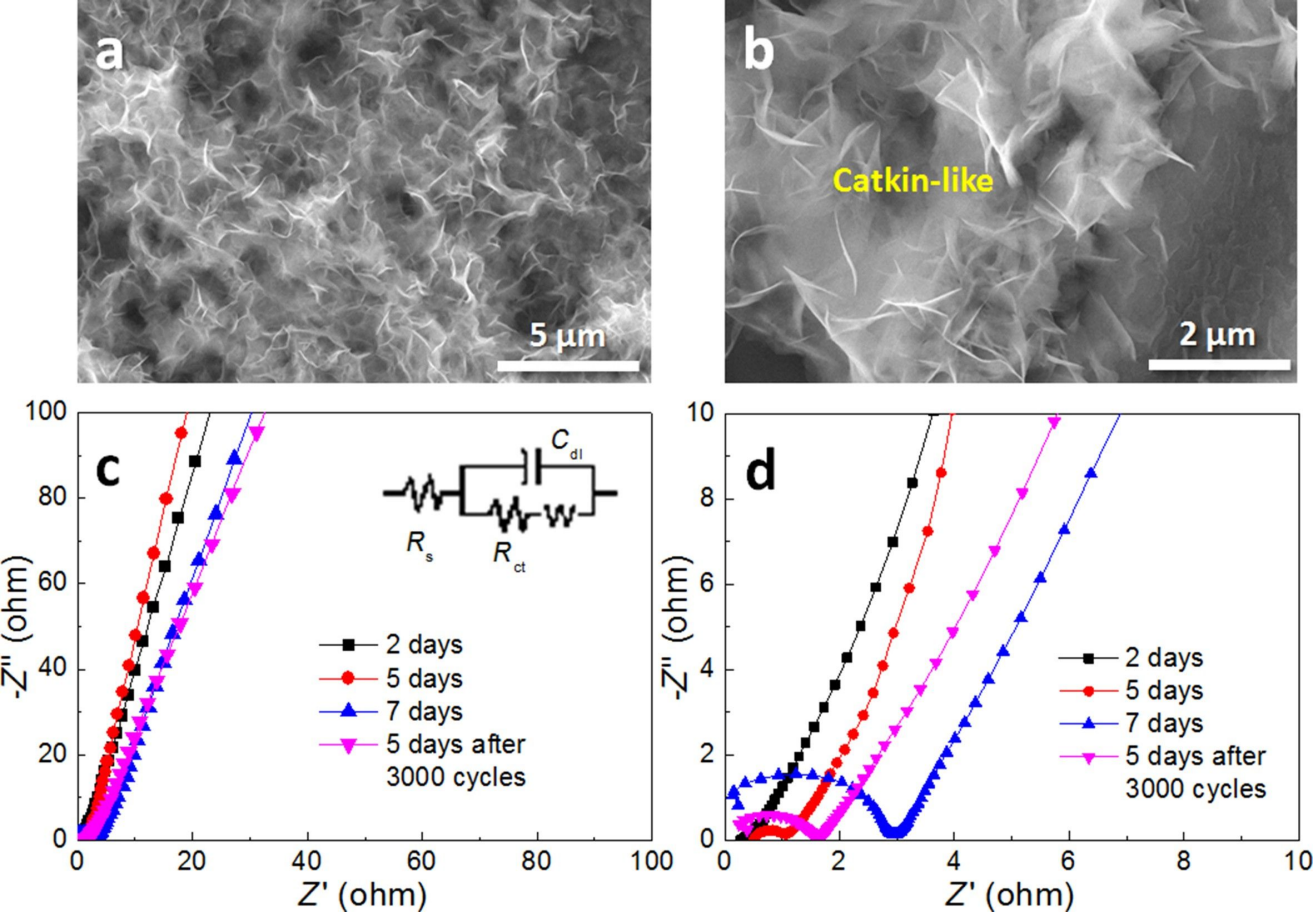
(a, b) Low- and high-magnification SEM images of Ni(OH)_2_/Ni-NF/MG-5 after a 3000 cycles at a GCD current density of 1 A/cm^3^; (c, d) Nyquist plots of Ni(OH)_2_/Ni-NF/MG-2, Ni(OH)_2_/Ni-NF/MG-5, Ni(OH)_2_/Ni-NF/MG-7 and Ni(OH)_2_/Ni-NF/MG-5 after 3000 cycles at a GCD current density of 1 A/cm^3^.

In order to explain the decline in the cycling performance of Ni(OH)_2_/Ni-NF/MG-5, the microstructure of the electrode after 3000 cycles was observed by SEM (Figure 7a,b). It is found that Ni(OH)_2_ nanopetals become thicker and interweave into a catkin-like morphology. Though a certain amount of nanopetals still remains, the “ion reservoir” structure is badly damaged. This results in a decrease in the surface area of active materials and subsequently leads to the decline in the volumetric capacitance of the electrode.

### Impedance spectroscopy

To better understand the kinetics of the charge transfer within the electrodes, EIS measurements were also carried out. The impedance spectra (Nyquist plots) are shown in Figure 7c,d. The inset is the equivalent electrical circuit. The intersection with the *Z*′-axis represents the equivalent series resistance (*R*_s_). All Nyquist plots exhibit a small semi-circle at high frequencies and a straight line at low frequencies. The semicircle represents the charge transfer impedance (*R*_ct_) for the redox reaction of Ni(OH)_2_/NiOOH at the electrode/electrolyte interface. The straight line at low frequencies indicates the diffusive resistance of the electrolyte ions (Warburg impedance) [49]. The simulated *R*_s_ values of the three electrodes are 1.27, 1.48 and 2.87 Ω/cm^2^. Moreover, the Ni(OH)_2_/Ni-NF/MG-2 exhibits the smallest *R*_ct_ value of 0.011 Ω/cm^2^, indicating its lowest charge transfer resistance. This is why Ni(OH)_2_/Ni-NF/MG-2 exhibits the best rate performance, whereas Ni(OH)_2_/Ni-NF/MG-7 exhibits the worst (*R*_ct_ = 0.028 Ω/cm^2^). Besides, Ni(OH)_2_/Ni-NF/MG-5 with the maximum slope at low frequency has the fastest ion diffusion rate among the three electrodes. It is found that both charge transfer resistance and ion diffusion resistance of the Ni(OH)_2_/Ni-NF/MG-5 increase after 3000 cycles, which is related to the agglomeration of Ni(OH)_2_ nanopetals.

### Performance of energy storage devices

**Figure 8:**
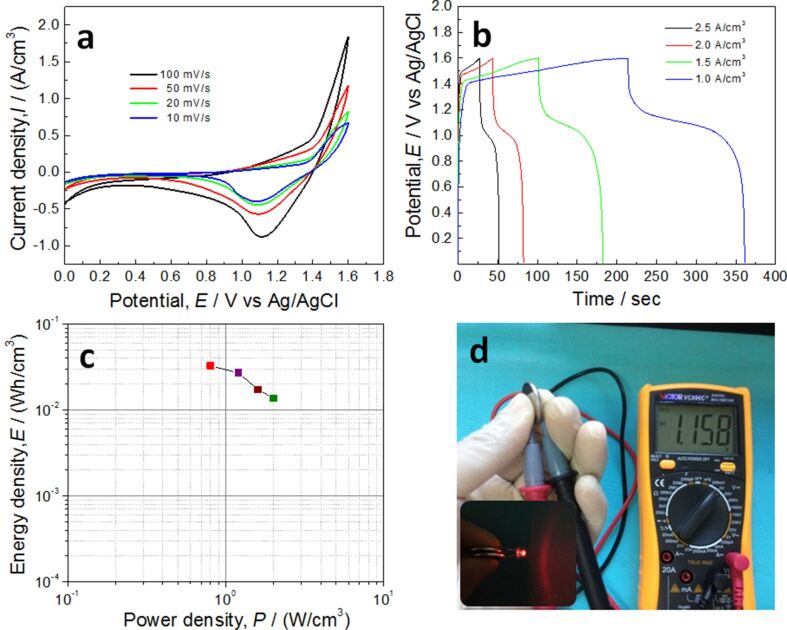
(a) CV curves and (b) GCD curves of a single SC device; (c) Ragone plot of the SC device; (d) voltage of a single fully charged device and a red LED bulb powered by two SC.

In order to further show the good energy storage performance of the as-prepared sandwich-like electrodes, the SC devices were assembled using Ni(OH)_2_/Ni-NF/MG-7. The CV and GCD curves of a single SC device are shown in Figure 8. CV and GCD curves present a higher voltage window of ca. 1.6 V. The calculated Ragone plot of a single SC device according to Equation 3 and Equation 4 is shown in Figure 8c. The volumetric energy densities are 32.7, 27.1, 17.3, 13.7 mWh/cm^3^ at power densities of 0.8, 1.2, 1.6, 2.0 W/cm^3^, respectively. The highest energy density in this work is comparable to that of a Ni@Ni(OH)_2_//graphene-CNT hybrid SC device (33.9 mWh/cm^3^ at a power density of 0.2 W/cm^3^) [50] and better than that of our previous NiO/np-Ni/MG symmetric supercapacitor device (19.82 mWh/cm^3^ at 0.4 W/cm^3^) [51]. This volumetric energy density is approximately three times larger than that of a thin-film lithium ion battery (1–12 mW h/cm^3^, 4 V/500 μAh) [52] and far exceeds that of a MnO_2_-Ni(OH)_2_/AB//active carbon asymmetric supercapacitor (3.62 mWh/cm^3^ at 11 mW/cm^3^) [39] and a NiCo-LDH//AC asymmetric capacitor (7.4 mWh/cm^3^ at 103 mW/cm^3^) [47]. After being fully charged, the voltage of a single SC device was measured with a digital multimeter. After a slow drop to 1.158 V, the voltage remains constant, as shown in Figure 8d. The two SC devices connected in series could power a red LED (1.8 V and 20 mA, inset of Figure 8d) for more than 2 min, which indicates that the as-prepared electrodes have a good energy storage performance.

Table 1 presents a summary of several electrode performance values compared with previous literature [40–42,46,48,53–55]. Although the cycling stability of the Ni(OH)_2_/Ni-NF/MG electrode is not comparable to that of Ni(OH)_2_/NF [41] and 2D MoSe_2_-Ni(OH)_2_ composite electrodes [46], the rate capability of Ni(OH)_2_/Ni-NF/MG electrode is greater than that of the two latter electrodes. (The capacitance retention rate still reaches 87.5% with 24-fold increase in current density). Except for the higher rate capability of NiCo_2_S_4_@Ni(OH)_2_ composite electrode with lower cycling stability [48], the present Ni(OH)_2_/Ni-NF/MG electrodes, which were obtained by a much easier environmentally friendly and cost-effective method, exhibit comparable or much better electrochemical performance among the electrodes based on electro-active Ni(OH)_2_. Besides, the key point is that Ni(OH)_2_/Ni-NF/MG electrodes exhibit excellent flexibility, which is a prominent feature of Ni(OH)_2_/Ni-NF/MG electrodes and meets the requirements of wearable devices. The outstanding electrochemical performance of Ni(OH)_2_/Ni-NF/MG electrodes can be ascribed to the following analysis of its unique sandwich-like electrode structure: Firstly, the conductive ligaments of the 3D continuous Ni nanofoam together with Ni-based MG substrate can provide multidimensional electron and ion transport pathways during the reversible faradic redox processes. Remarkably, the Ni(OH)_2_/Ni-NF/MG electrodes demonstrate an excellent flexibility and they can be bent into a circle with a diameter of ca. 5 mm and even be tied into a small bowknot, which indicates the integrated internal structure of the electrode. Secondly, the squiggly interconnected nanopetals grown on the 3D Ni nanofoam can play the role of an “ion reservoir”, yielding fast ion transfer, short ion transport distances and sufficient contact at active material/electrolyte interfaces. Finally, a complete integrated electrode is formed by the close bonding between the Ni(OH)_2_ nanopetals and the Ni nanofoam substrate, avoiding the addition of conductive agent and binder, resulting in highly efficient electron transfer and ion transport. Accordingly, the sandwich-like Ni(OH)_2_/Ni-NF/MG electrodes with good energy storage performance, high cycling stability as well as excellent flexibility are a promising prospect in wearable energy storage devices.

**Table 1:** Comparison of Ni(OH)_2_/Ni-NF/MG with other electrode materials.

electrodes	preparation method	*C*_sp_^a^	rate capability	cycling stability	ref.

3D-porous Ni(OH)_2_	solvothermal precipitation	2110 F/g at 1.0 A/g	55.5%, 10-fold	2000 cycles at 5 A/g, 53% retention	[40]
Ni(OH)_2_/NF^b^	hydrothermal	3.51 F/cm^2^ at 2 mA/cm^2^	46.2%, 20-fold	7500 cycles at 20 mA/cm^2^, 95.5% retention	[41]
Ni(OH)_2_ nanosheets	hydrothermal	609.2 C/g at 1 A/g	62.4%, 20-fold	1000 cycles at 5 A/g, 83.3% retention	[42]
2D MoSe_2_-Ni(OH)_2_	hydrothermal	1175 F/g at 1 A/g	85.6%, 10-fold	3000 cycles at 2 A/g, 90% retention	[46]
NiCo_2_S_4_@Ni(OH)_2_	hydrothermal and electrodeposition	680 F/g at 5 mA/cm^2^	94.9%, 20-fold	2000 cycles at 40 mA/cm^2^, 81.4% retention	[48]
Ni(OH)_2_@ACMT^c^	acid treatment and hydrothermal	1568 F/g at 1 A/g	51.1%, 20-fold	3000 cycles at 5 A/g, 84.3% retention	[53]
Ni(OH)_2_@NF^b^	hydrothermal	693 F/g at 4 A/g	34.8%, 3-fold	3000 cycles at 10 A/g, 77.3% retention	[54]
CNT^d^@Ni(OH)_2_	chemical bath deposition	1136 F/g at 2 A/g	33.8%, 10-fold	1000 cycles at 8 A/g, 92% retention	[55]
Ni(OH)_2_/Ni-NF^e^/MG^f^	dealloying and water-immersion	822.6 F/cm^3^ at 0.5 A/cm^3^	87.5%, 24-fold	3000 cycles at 1 A/cm^3^, 83.7% retention	this work

^a^specific capacitance; ^b^Ni foam; ^c^acid-treated carbon microtubes; ^d^carbon nanotubes; ^e^nanofoam; ^f^metallic glass.

## Conclusion

In summary, a sandwich-like Ni(OH)_2_/Ni-NF/MG electrode with good flexibility was synthesized through a two-step synthesis including the dealloying of ductile Ni_40_Zr_20_Ti_40_ metallic glass to form a Ni nanofoam interlayer and subsequent immersion in water to create a Ni(OH)_2_ nanopetal network on the Ni nanofoam surface. The Ni(OH)_2_ nanopetals interweave with each other and grow vertically on the surface of Ni nanofoam to create an “ion reservoir”, which facilitates the ion diffusion in the electrode reaction. Because of this unique structure, the Ni(OH)_2_/Ni-NF/MG-7 electrode reveals a high volumetric capacitance of 966.4 F/cm^3^ at a current density of 0.5 A/cm^3^ and the Ni(OH)_2_/Ni-NF/MG-5 electrode exhibits an excellent cycling stability (83.7% of the initial capacity after 3000 cycles at a current density of 1 A/cm^3^). In addition, symmetric supercapacitor (SC) devices were assembled using Ni(OH)_2_/Ni-NF/MG-7 and showed a maximum volumetric energy density of ca. 32.7 mWh/cm^3^ at a power density of 0.8 W/cm^3^, and of 13.7 mWh/cm^3^ when the power density increased to 2 W/cm^3^. We proved for the first time that Ni(OH)_2_ nanopetals were successfully prepared without elevated temperatures and nickel salt additives. This work may provide with a new idea for the synthesis of nanostructured Ni(OH)_2_ by a simple and environmentally friendly approach.
